# Efficacy of a novel topical combination of esafoxolaner, eprinomectin and praziquantel against *Ixodes ricinus* and *Ixodes scapularis* in cats

**DOI:** 10.1051/parasite/2021019

**Published:** 2021-04-02

**Authors:** Joe Prullage, Anthony Pfefferkorn, Martin Knaus, Justin Frost, Elizabeth Mitchell, Eric Tielemans

**Affiliations:** 1 Boehringer-Ingelheim Animal Health, Missouri Research Center 6498 Jade Rd. Fulton MO 65251 USA; 2 Boehringer-Ingelheim Animal Health 29 Avenue Tony Garnier 69007 Lyon France; 3 Boehringer-Ingelheim Vetmedica GmbH, Kathrinenhof Research Center Walchenseestr. 8–12 83101 Rohrdorf Germany; 4 Boehringer-Ingelheim Animal Health 1730 Olympic Drive Athens GA 30601 USA

**Keywords:** Cat, *Ixodes ricinus*, *Ixodes scapularis* esafoxolaner, Efficacy

## Abstract

Esafoxolaner is a purified enantiomer of afoxolaner with insecticidal and acaricidal properties. It is combined with eprinomectin and praziquantel in a novel topical endectoparasiticide formulation for cats. The efficacy of this novel formulation was evaluated in three *Ixodes ricinus* and two *Ixodes scapularis* experimental studies, with comparable designs. In each study, cats were randomly allocated, based on a pre-treatment tick infestation and count, to a placebo control group or a group treated with the minimum recommended dose of the novel formulation. Cats were infested two days before treatment and weekly thereafter. Immediate efficacy was evaluated 48 h after treatment; persistent efficacy was evaluated 48 h after new weekly infestations for at least one month after the treatment (in one of the studies, the first two weeks of persistent efficacy against *I. ricinus* were not tested). Efficacy was calculated at each timepoint by comparison of arithmetic means of live ticks found in the control and the treated groups. In the three studies targeting *I. ricinus*, immediate and persistent efficacies ranged between 91% and 100% for five weeks. In the two studies targeting *I. scapularis*, immediate and persistent efficacies ranged between 95% and 100%, and 98% and 100% for one month, respectively. These studies provide robust evidence of efficacy of the novel topical formulation of esafoxolaner, eprinomectin and praziquantel against experimental *I. ricinus* and *I. scapularis* infestations for at least one month in cats.

## Abstract (fr)

L’esafoxolaner est un énantiomère purifié de l’afoxolaner aux propriétés insecticides et acaricides. Il est associé à l’éprinomectine et au praziquantel dans une nouvelle formulation d’endectoparasiticide topique pour chats. L’efficacité de cette nouvelle formulation a été évaluée dans trois études expérimentales sur *Ixodes ricinus* et deux sur *Ixodes scapularis*, avec des conceptions comparables. Dans chaque étude, les chats ont été répartis au hasard, sur la base d’une infestation et d’un nombre de tiques avant le traitement, dans un groupe témoin placebo ou dans un groupe traité avec la dose minimale recommandée de la nouvelle formulation. Les chats ont été infestés deux jours avant le traitement et une fois par semaine par la suite. L’efficacité immédiate a été évaluée 48 heures après le traitement et l’efficacité persistante a été évaluée 48 heures après les nouvelles infestations hebdomadaires pendant au moins un mois après le traitement (dans l’une des études, les deux premières semaines d’efficacité persistante contre *I. ricinus* n’ont pas été testées). L’efficacité a été calculée à chaque temps d’évaluation par comparaison des moyennes arithmétiques des tiques vivantes trouvées dans les groupes témoins et traités. Dans les trois études ciblant *I. ricinus*, les efficacités immédiates et persistantes variaient entre 91 % et 100 % pendant cinq semaines. Dans les deux études ciblant *I. scapularis*, les efficacités immédiates et persistantes variaient respectivement entre 95 % et 100 % et 98 % et 100 % pendant un mois. Ces études fournissent des preuves solides de l’efficacité de la nouvelle formulation topique d’esafoxolaner, d’éprinomectine et de praziquantel contre les infestations expérimentales par *I. ricinus* et *I. scapularis* pendant au moins un mois chez le chat.

## Introduction

*Ixodes ricinus* is the most common tick species infesting companion animals and is present throughout Europe, especially in central Europe [5, 6, 11, 34, 44]. *Ixodes scapularis* is among the main tick species infesting cats in North America [7, 13, 23, 45]. *Ixodes spp.* infestations are an important concern for pet owners and public health [1, 4, 12, 35, 36]. In addition to causing local discomfort and local inflammatory reactions, they may also transmit pathogens that can cause severe disease in animals and humans such as anaplasmosis, babesiosis and borreliosis [8, 9, 15, 17, 19, 21, 26, 27, 37–42]. Tick prevalence, as well as the risk for transmission of tick-borne diseases, has been increasing over the last three decades in both Europe and the United States [16, 18, 22, 28, 29, 31].

A novel topical combination of (S)-afoxolaner, eprinomectin and praziquantel has been designed to offer a wide spectrum of antiparasitic activities. Esafoxolaner is the active purified (S)-enantiomer of afoxolaner, the racemic mixture. Afoxolaner has already been proven effective in dogs against several Ixodidae*,* including *I. ricinus* and *I. scapularis* when administered orally as a single-compound (Nexgard^®^) or combination product (Nexgard^®^ Spectra) [14, 20, 24, 25, 32, 33].

The objectives of the four studies presented here were to assess the efficacy of this novel formulation for the treatment and control of *I. ricinus* and *I. scapularis* infestations, when administered topically to cats at the minimum recommended label dose in a laboratory setting.

## Materials and methods

### Ethics

The study protocols were reviewed and approved by the Sponsor’s and Investigator’s Institutional Animal Care and Use Committees or by the responsible governmental authority as per legal requirements. Cats were managed and handled similarly within each study and with due regard for their wellbeing.

### Study designs

The studies were designed in accordance with the “World Association for the Advancement of Veterinary Parasitology (W.A.A.V.P.) guidelines for evaluating the efficacy of parasiticides for the treatment, prevention and control of flea and tick infestation on dogs and cats” [30]. The three *I. ricinus* studies were also designed in accordance with the Committee for Medicinal Products for Veterinary Use (CVMP) “Guideline for the Testing and Evaluation of the Efficacy of Antiparasitic Substances for the Treatment and Prevention of Tick and Flea Infestation in Dogs and Cats”, EMEA/CVMP/EWP/005/2000-Rev.3. Two of the *I. ricinus* studies and the two *I. scapularis* studies were conducted in accordance with Good Clinical Practices as described in “International Cooperation on Harmonization of Technical Requirements for Registration of Veterinary Medicinal Products (VICH) guideline GL9”.

Each study was conducted using a randomized design based on a pre-treatment parasite infestation and count, for allocation to either a placebo/untreated negative control group or a novel formulation treated group. Personnel collecting animal health and efficacy data were blinded to treatment group. Cats were single housed during the periods of tick infestation/tick removal. The efficacy assessment was based on comparison of live ticks found in the control versus the treated group at identical time-points after treatment on Day 0. All efficacy assessments were performed 48 h after treatment (immediate efficacy) or subsequent infestations (persistent efficacy).

The five studies were conducted at different locations, *I. ricinus* studies in Europe and *I. scapularis* studies in the United States. Individual specifics of the studies are detailed in Tables 1 and 2.

**Table 1:** Regulatory context, location, timing and animals by study.

Study #	Regulatory status	Test site location	Date of experimental phase	Animals^1^		
				Number and sex	Age (months)	Bodyweight (kg)
*I. ricinus #1*	GCP	Germany, EU	Nov–Dec 2015	6 male, 6 female	16–34	2.6–5.3
*I. ricinus #2*	Exploratory	Germany, EU	Jul–Aug 2016	8 male, 8 female	10–25	2.6–5.8
*I. ricinus #3*	GCP	France, EU	May–Jul 2017	10 male, 10 female	6–8	2.7–5.7
*I. scapularis #1*	GCP	Missouri, USA	Jul–Sep 2017	8 male, 12 female	20–59	3.0–6.2
*I. scapularis #2*	GCP	Georgia, USA	Jan–Feb 2018	11 male, 9 female	8–12	2.2–5.3

1 All cats were Domestic Long/Short-Hair, healthy and purpose-bred.

**Table 2:** Tick colonies (origin, number and sex), infestations and tick counts for efficacy evaluations by study.

Study #	Ixodes strain origin	Number of ticks at infestation	Infestation timepoints	Efficacy timepoints (48 h after treatment or infestation)
*I. ricinus #1*	North and southwest Germany, EU	40 females^1^	Days −2, 7, 14, 21, 28, 35	Days 2, 9, 16, 23, 30, 37
*I. ricinus #2*	Berlin, Germany, EU	40 females^2^ (25 at Day 42)	Days −2, 21, 28, 35, 42	Days 2, 23, 30, 37, 44
*I. ricinus #3*	North and southwest Germany, EU	50 females^2^	Days −2, 8, 14, 20, 28, 35	Days 2, 10, 16, 22, 30, 37
*I. scapularis #1*	Oklahoma, USA	50 (males and females)	Days −2, 7, 14, 21, 30	Days 2, 9, 16, 23, 32
*I. scapularis #2*	South Carolina, USA^1^	50 (males and females)	Days −2, 7, 14, 21, 30	Days 2, 9, 16, 23, 32

1 Ticks wild caught from the field.

2 And an undefined number of males.

### Animals

The cats were healthy, purpose-bred laboratory Domestic Short-hair cats. Sex, age and bodyweight are described in Table 1.

### Parasites

Ticks used in all *I. ricinus* studies and in one of the *I. scapularis* studies originated from laboratory-maintained colonies genetically enriched at least every 5 years, not previously exposed and not known to be resistant to any ectoparasiticide compound, and were pathogen-free. Ticks used in the second *I. scapularis* study were collected from the wild. The origin of the ticks is described in Table 2.

### Treatment

Cats were treated once on Day 0. The treatment was applied as one spot directly on the skin, after parting the hair, in the midline of the neck between the base of the skull and the shoulder blades. Cats assigned to the placebo control group were treated with mineral oil at 0.12 mL/kg (in one of the *I. ricinus* studies, cats were sham dosed). Cats assigned to the novel formulation group were treated with the minimum recommended dose of 0.12 mL/kg, delivering 1.44 mg/kg esafoxolaner, 0.48 mg/kg eprinomectin and 10.0 mg/kg praziquantel. The formulation used in the first of the *I. ricinus* studies was an early-stage experimental formulation with identical concentration and composition of active ingredients, but with some differences in the solvent system. The formulation used in the other four studies was the final novel formulation.

Health observations were conducted daily and at hourly intervals for 4 h after treatment in all studies to detect any adverse reaction or health abnormality.

### Tick infestations and counts

The timepoints of infestation, count and the number of ticks used at each individual infestation are described in Table 2. In the five studies, ticks were placed on the side or the head of each cat, avoiding the application site. To minimize grooming and scratching of ticks, cats were fitted with Elizabethan collars (except in the second *I. ricinus* study) at the time of infestation. The collars were removed before treatment on Day 0, or when ticks were counted on Day 9 and afterwards. In the *I. ricinus* studies, all cats were sedated for infestations and counts; in the *I. scapularis* studies, cats were only sedated when necessary.

The whole body was examined for tick counts. The hair was pushed manually against its natural nap to expose the ticks. The whole body was thoroughly searched. All ticks found were removed using a tick extractor. After the visual and digital examination was complete, the hair coat was thoroughly combed using a fine-toothed flea comb for secondary confirmation that all ticks had been found and counted. The live or dead status was determined. In one of the *I. ricinus* studies, the attached or free status of each collected tick was also determined and any tick found in a comb was classified by default as attached. In the *I. scapularis* studies, dead ticks found in the enclosures were also counted.

### Statistical analysis

The efficacy variable was the live tick count (or live attached tick count in one of the *I. ricinus* studies). Arithmetic means were calculated for each treatment group at each efficacy timepoint. The percent efficacy was calculated as [(*C* − *T*)/*C*] × 100, where *C* was the arithmetic mean of the counts among the control cats and *T* was the arithmetic mean among the treated cats.

To compare the groups statistically, the logarithm of (counts + 1) was analyzed. The log-counts of each treated group were compared to the log-counts of the control group using an *F*-test adjusted for the allocation blocks used to randomize the animals to the treatment groups at each timepoint separately. The MIXED procedure in SAS Version 9.4 was used for the analysis, with group listed as a fixed effect and the allocation blocks listed as a random effect. All statistical comparisons used a 5% significance level.

## Results

### Efficacy against *I. ricinus*

The efficacy results obtained in *I. ricinus* studies are summarized in Table 3.

**Table 3:** *Ixodes ricinus* studies: mean live tick counts per group and efficacy results.

Study	*n*	Arithmetic means (AM) of live ticks and % efficacy^1^ on indicated days							
*I. ricinus* #1			Day 2	Day 9	Day 16	Day 23	Day 30	Day 37	Day 44
Control group^2^	6	AM	20.5	20.8	19.0	24.3	31.2	30.8	–
Treated group^4^	6	AM	1.8	0.0	0.0	0.0	0.3	2.7	–
		% efficacy^5^	91.1	100.0	100.0	100.0	98.9	91.4	–
*I. ricinus* #2			Day 2	Day 9	Day 16	Day 23	Day 30	Day 37	Day 44
Control group^3^	8	AM	23.1	–	–	27.0	29.5	26.6	14.4
Treated group^4^	8	AM	1.9	–	–	0.4	0.0	0.0	0.9
		% efficacy^5^	91.9	–	–	98.6	100.0	100.0	93.9
*I. ricinus* #3			Day 2	Day 10	Day 16	Day 22	Day 30	Day 37	–
Control group^3^	10	AM	21.0	17.8	18.1	23.9	19.1	21	–
Treated group^4^	10	AM	0.0	0.0	0.0	0.0	0.2	0.0	–
		% efficacy^5^	100.0	100.0	100.0	100.0	99.0	100.0	–

*Note*: *p*-values^6^ for comparison of the untreated and treated groups = <0.0001 at all time-points.

*n* = number of cats per group.

1 The *I. ricinus* counts were analyzed using SAS Version 9.4. The logarithm of the (count + 1) was analyzed at each study day separately using the MIXED procedure with treatment group used as a fixed effect and blocks used as a random effect.

2 Control group = Mineral sham dosed.

3 Control group = Mineral oil administered topically once on Day 0 at 0.12 mL/kg.

4 Treated group = Novel formulation administered topically once on Day 0 at 0.12 mL/kg, providing 1.4 mg/kg esafoxolaner, 10.0 mg/kg praziquantel and 0.5 mg/kg eprinomectin.

5 Percent efficacy = [(*C* – *T*)/*C*] × 100, where *C* and *T* are the arithmetic means of the control and treated groups, respectively.

6 Two-sample probability value comparing the population means of the treated group vs. the control group.

In the *I. ricinus* studies, the curative efficacies of the novel formulation, 48 h after treatment, were 91.1%, 91.9% and 100%, respectively; the preventive efficacies 48 h after subsequent infestations were at least 91.4% for five weeks, 93.9% for six weeks, and 100.0% for five weeks, respectively. At each timepoint and in the three studies, the number of live ticks was significantly lower on treated animals than on control animals (*p* < 0.0001).

In all three studies and considering all timepoints, mean tick retention in the control animals ranged from 36% to 78%, demonstrating robustness of the challenge.

### Efficacy against *I. scapularis*

The percent efficacy results obtained in studies *I. scapularis* #1 and #2 are summarized in Table 4.

**Table 4:** *Ixodes scapularis* studies: mean live tick counts per group and efficacy results

Study	*n*	Arithmetic means (AM) of live ticks and % efficacy^1^ on indicated days					
*I. scapularis* #1			Day 2	Day 9	Day 16	Day 23	Day 32
Control group^2^	8	AM	26.5	27.9	30.4	32.1	32.5
Treated group^3^	8	AM	1.3	0.2	0.0	0.4	0.0
		% efficacy^4^	95.1	99.3	100.0	98.8	100.0
*I. scapularis* #2			Day 2	Day 9	Day 16	Day 22	Day 32
Control group^2^	10	AM	24.5	26.5	27.2	24.4	22.2
Treated group^3^	10	AM	0.3	0.0	0.0	0.1	0.4
		% efficacy^4^	98.8	100.0	100.0	99.6	98.2

*Note*: *p*-values^5^ for comparison of the untreated and treated groups = <0.0001 at all time-points.

*n* = number of cats per group.

1 The *I. scapularis* counts were analyzed using SAS Version 9.4. The logarithm of the (count + 1) was analyzed at each study day separately using the MIXED procedure with treatment group used as a fixed effect and blocks used as a random effect.

2 Control group = Mineral oil administered topically once on Day 0 at 0.12 mL/kg

3 Treated group = Novel formulation administered topically once on Day 0 at 0.12 mL/kg, providing 1.4 mg/kg esafoxolaner, 10.0 mg/kg praziquantel and 0.5 mg/kg eprinomectin

4 Percent efficacy = [(*C* – *T*)/*C*] × 100, where *C* and *T* are the arithmetic means of the control and treated groups, respectively.

5 Two-sample probability value comparing the population means of the treated group vs. the control group.

The curative efficacy of the novel formulation, 48 h after treatment, was 95.1% and 98.8% in the two studies. The preventive efficacy 48 h after subsequent infestations was at least 98.8% for one month in the first and was at least 98.2% for one month in the second study. At each timepoint and in both studies, the number of live ticks was significantly lower on treated animals than on control animals (*p* < 0.0001). Significantly fewer dead ticks were found in the control animals in both studies, (*p* ≤ 0.0005 and *p* ≤ 0.02, respectively). In both studies and considering all timepoints, mean tick retention in the control animals ranged from 44% to 65%, demonstrating robustness of the challenge.

No adverse reactions related to treatment were observed in any of the five studies.

## Discussion

Companion animals may contribute to the circulation and spread in the environment of ticks, fleas, and tick and flea-borne pathogens, and require control and protection by year-round use of ectoparasiticides with both insecticidal and acaricidal activity [2, 12, 43, 45].

The results of these five studies illustrate the high level of efficacy of the novel topical formulation of esafoxolaner, eprinomectin and praziquantel against *I. ricinus* and *I. scapularis* infestations. This high level of efficacy is achieved within 48 h of treatment and is maintained within 48 h following infestation for at least one month after application.

The control of multiple and various concurrent parasitic infestations by a range of cat parasites is important for cats but also public health [6, 10, 46]. In a European survey on domestic cats, co-infestation with endo- and ectoparasites was observed in 14% of the subjects [3]. This novel formulation offers a broad spectrum of efficacy against the main parasites of cats including ecto- and endoparasites.

In addition to a high level of efficacy and safety, owner compliance with a regular treatment schedule is an important feature for successful control of ticks. The simple conditions of use and of treatment application of this product should ensure a high level of compliance.

This novel formulation provides pet owners and veterinarians with an effective solution for an integrated approach for cats presenting multiple parasitic infestations, or presenting risks associated with these parasitic infestations.

## Competing interest

The work reported herein was funded by Boehringer-Ingelheim. The authors are current employees of Boehringer-Ingelheim. Other than this, the authors declare no conflict of interest. This document is provided for scientific purposes only. Any reference to a brand or trademark herein is for information purposes only and is not intended for any commercial purposes or to dilute the rights of the respective owners of the brand(s) or trademark(s).

NexGard^®^ is a registered trademark of the Boehringer-Ingelheim Group.

## References

[1] Berrada ZL, Telford SR 3rd. 2009. Burden of tick-borne infections on American companion animals. Topics in Companion Animal Medicine, 24(4), 175–181. 19945085 10.1053/j.tcam.2009.06.005 PMC2802828

[2] Beugnet F. 2013. Guide to vector borne diseases of pets. France: Ed Ferreol, 425 p.

[3] Beugnet F, Bourdeau P, Chalvet-Monfray K, Cozma V, Farkas R, Guillot J, Halos L, Joachim A, Losson B, Miró G, Otranto D, Renaud M, Rinaldi L. 2014. Parasites of domestic owned cats in Europe: Co-infestations and risk factors. Parasites & Vectors, 7, 291. 24965063 10.1186/1756-3305-7-291 PMC4082530

[4] Beugnet F, Halos L. 2015. Parasitoses & vector borne diseases of cats. Beugnet F, Halos L, Scientific Editors. Ferreol: Lyon, France. 381 p. ISBN 978-2-9550805-0-4.

[5] Beugnet F, Halos L, Guillot J. 2018. Chapter: Tick Infestation. Textbook of Clinical Parasitology in dogs and cats. France: Grupo Asis. p. 236–249.

[6] Beugnet F, Marié JL. 2009. Emerging arthropod-borne diseases of companion animals in Europe. Veterinary Parasitology, 163, 298–305. 19403239 10.1016/j.vetpar.2009.03.028

[7] Blagburn BL, Dryden MW. 2009. Biology, treatment, and control of flea and tick infestations. Veterinary Clinics of North America: Small Animal Practice, 39(6), 1173–1200. 10.1016/j.cvsm.2009.07.001 19932369

[8] Claerebout E, Losson B, Cochez C, Casaert S, Dalemans AC, De Cat A, Madder M, Saegerman C, Heyman P, Lempereur L. 2013. Ticks and associated pathogens collected from dogs and cats in Belgium. Parasites & Vectors, 19(6), 183. 10.1186/1756-3305-6-183 PMC3688525 23777784

[9] Davies S, Abdullah S, Helps C, Tasker S, Newbury H, Wall R. 2017. Prevalence of ticks and tick-borne pathogens: *Babesia* and *Borrelia* species in ticks infesting cats of Great Britain. Veterinary Parasitology, 15(244), 129–135. 10.1016/j.vetpar.2017.07.033 28917304

[10] Deplazes P, van Knapen F, Schweiger A, Overgaauw PA. 2011. Role of pet dogs and cats in the transmission of helminthic zoonoses in Europe, with a focus on echinococcosis and toxocarosis. Veterinary Parasitology, 182, 41–53. 21813243 10.1016/j.vetpar.2011.07.014

[11] Dobson AD, Taylor JL, Randolph SE. 2011. Tick (*Ixodes ricinus*) abundance and seasonality at recreational sites in the UK: hazards in relation to fine-scale habitat types revealed by complementary sampling methods. Ticks and Tick Borne Diseases, 2(2), 67–74. 21771540 10.1016/j.ttbdis.2011.03.002

[12] Dryden MW, Hodgkins E. 2010. Vector-borne diseases in pets: the stealth health threat. Compendium on Continuing Education, 32, E1–E4. 20949426

[13] Dryden MW, Payne PA. 2004. Biology and control of ticks infesting dogs and cats in North America. Veterinary Therapeutics, 5, 139–154. 15468011

[14] Dumont P, Blair J, Fourie J, Chester T, Larsen D. 2014. Evaluation of the efficacy of afoxolaner against two European dog tick species: *Dermacentor reticulatus* and *Ixodes ricinus*. Veterinary Parasitology, 201, 216–219. 24629424 10.1016/j.vetpar.2014.02.017

[15] Egyed L, Elö P, Sréter-Lancz Z, Széll Z, Balogh Z, Sréter T. 2012. Seasonal activity and tick-borne pathogen infection rates of *Ixodes ricinus* ticks in Hungary. Ticks and Tick Borne Diseases, 3, 90–94. 22445929 10.1016/j.ttbdis.2012.01.002

[16] Estrada-Peña A, de la Fuente J. 2014. The ecology of ticks and epidemiology of tick-borne viral diseases. Antiviral Research, 108, 104–128. 24925264 10.1016/j.antiviral.2014.05.016

[17] Geurden T, Becskei C, Six R, Maeder S, Latrofa M, Otranto D, Farkas R. 2018. Detection of tick-borne pathogens in ticks from dogs and cats in different European countries. Ticks and Tick Borne Disease, 9(6), 1431–1436. 10.1016/j.ttbdis.2018.06.013 29983263

[18] Gray JS, Dautel H, Estrada-Peña A, Kahl O, Lindgren E. 2009. Effects of climate change on ticks and tick-borne diseases in Europe. Interdisciplinary Perspectives on Infectious Diseases, 2009, 12. Article ID 593232. 10.1155/2009/593232 PMC2648658 19277106

[19] Gray JS, von Stedingk LV, Gürtelschmid M, Granström M. 2002. Transmission Studies of *Babesia microti* in *Ixodes ricinus* ticks and gerbils. Journal of Clinical Microbiology, 40(4), 1259–1263. 11923342 10.1128/JCM.40.4.1259-1263.2002 PMC140393

[20] Halos L, Lebon W, Chalvet-Monfray K, Larsen D, Beugnet F. 2014. Immediate efficacy and persistent speed of kill of a novel oral formulation of afoxolaner (NexGard^TM^) against induced infestations with *Ixodes ricinus* ticks. Parasites & Vectors, 5, 452. 10.1186/1756-3305-7-452 PMC4262065 25261196

[21] Hoyt K, Chandrashekar R, Beall M, Leutenegger C, Lappin M. 2018. Evidence for Clinical Anaplasmosis and Borreliosis in Cats in Maine. Topics in Companion Animal Medicine, 33(2), 40–44. 30223986 10.1053/j.tcam.2018.05.002

[22] Jaenson TG, Jaenson DG, Eisen L, Petersson E, Lindgren E. 2012. Changes in the geographical distribution and abundance of the tick *Ixodes ricinus* during the past 30 years in Sweden. Parasites & Vectors, 5, 8. 22233771 10.1186/1756-3305-5-8 PMC3311093

[23] Keirans JE, Hutcheson HJ, Durden LA, Klompen JSH. 1996. Ixodes(Ixodes) scapularis (Acari: Ixodidae): redescription of all active stages, distribution, hosts, geographical variation, and medical and veterinary importance. Journal of Medical Entomology, 33, 297–318. 8667375 10.1093/jmedent/33.3.297

[24] Kondo Y, Kinoshita G, Drag M, Chester T, Larsen D. 2014. Evaluation of the efficacy of afoxolaner against *Haemaphysalis longicornis* on dogs. Veterinary Parasitology, 201, 229–231. 24629429 10.1016/j.vetpar.2014.02.019

[25] Kunkle B, Daly S, Drag M, Dumont P, Larsen D. 2014. Assessment of the efficacy of orally administered afoxolaner against *Rhipicephalus sanguineus* sensu lato. Veterinary Parasitology, 201, 226–228. 24629425 10.1016/j.vetpar.2014.02.018

[26] Lappin MR, Chandrashekar R, Stillman B, Liu J, Mather TN. 2015. Evidence of *Anaplasma phagocytophilum* and *Borrelia burgdorferi* infection in cats after exposure to wild-caught adult *Ixodes scapularis*. Journal of Veterinary Diagnosis and Investigation, 27(4), 522–525. 10.1177/1040638715593598 26179101

[27] Lappin MR. 2018. Update on flea and tick associated diseases of cats. Veterinary Parasitology, 254, 26–29. 29657007 10.1016/j.vetpar.2018.02.022

[28] Léger E, Vourc’h G, Vial L, Christine Chevillon C, McCoy KD. 2013. Changing distributions of ticks: causes and consequence. Experimental and Applied Acarology, 59, 219–244. 23015121 10.1007/s10493-012-9615-0

[29] Lindgren E, Tälleklint L, Polfeldt T. 2000. Impact of climatic change on the northern latitude limit and population density of the disease-transmitting European tick *Ixodes ricinus*. Environmental Health Perspectives, 108(2), 119–123. 10656851 10.1289/ehp.00108119 PMC1637900

[30] Marchiondo AA, Holdsworth PA, Fourie LJ, Rugg D, Hellmann K, Snyder DE, Dryden MW. 2013. World Association for the Advancement of Veterinary Parasitology (W.A.A.V.P.) second edition: Guidelines for evaluating the efficacy of parasiticides for the treatment, prevention and control of flea and tick infestations on dogs and cats. Veterinary Parasitology, 194, 84–97. 23741753 10.1016/j.vetpar.2013.02.003

[31] McPherson M, García-García A, Cuesta-Valero FJ, Beltrami H, Hansen-Ketchum P, MacDougall D, Ogden NH. 2017. Expansion of the lyme disease vector *Ixodes Scapularis* in Canada inferred from CMIP5 climate projections. Environmental Health Perspecive, 125(5), 057008. 10.1289/EHP57 PMC5730520 28599266

[32] Mitchell E, McCall JS, Chester T, Larsen D. 2014. Efficacy of afoxolaner against *Ixodes scapularis* ticks in dogs. Veterinary Parasitology, 201, 223–225. 24685321 10.1016/j.vetpar.2014.02.015

[33] Mitchell E, Dorr P, Everett WR, Chester T, Larsen D. 2014. Efficacy of afoxolaner against *Dermacentor variabilis* ticks in dogs. Veterinary Parasitology, 201, 220–222. 24629426 10.1016/j.vetpar.2014.02.016

[34] Ogden NH, Cripps P, Davison CC, Owen G, Parry JM, Timms BJ, Forbes AB. 2000. The *ixodid* tick species attaching to domestic dogs and cats in Great Britain and Ireland. Medical and Veterinary Entomology, 14(3), 332–338. 11016442 10.1046/j.1365-2915.2000.00244.x

[35] Otranto D, Dantas-Torres F, Breitschwerdt EB. 2009a. Managing canine vector-borne diseases of zoonotic concern: part one. Trends in Parasitology, 25, 157–163. 19269898 10.1016/j.pt.2009.01.003

[36] Otranto D, Dantas-Torres F, Breitschwerdt EB. 2009b. Managing canine vector-borne diseases of zoonotic concern: part two. Trends in Parasitology, 25, 228–235. 19346164 10.1016/j.pt.2009.02.005

[37] Persichetti M, Solano-Gallego L, Serrano L, Altet L, Reale S, Masucci M, Pennisi M. 2016. Detection of vector-borne pathogens in cats and their ectoparasites in southern Italy. Parasites & Vectors, 9(1), 247. 27160725 10.1186/s13071-016-1534-1 PMC4862052

[38] Savidge C, Ewing P, Andrews J, Aucoin D, Lappin M, Moroff S. 2016. *Anaplasma phagocytophilum* infection of domestic cats: 16 cases from the northeastern USA. Journal of Feline Medicine and Surgery, 18, 85–91. 25680735 10.1177/1098612X15571148 PMC11149011

[39] Schorn S, Pfister K, Reulen H, Mahling M, Manitz J, Thiel C, Silaghi C. 2011. Prevalence of *Anaplasma phagocytophilum* in *Ixodes ricinus* in Bavarian public parks, Germany. Ticks and Tick Borne Diseases, 2(4), 196–203. 22108012 10.1016/j.ttbdis.2011.09.009

[40] Schorn S, Pfister K, Reulen H, Mahling M, Silaghi C. 2011. Occurrence of *Babesia spp., Rickettsia spp.* and *Bartonella spp.* in *Ixodes ricinus* in Bavarian public parks, Germany. Parasites & Vectors, 4, 135. 21762494 10.1186/1756-3305-4-135 PMC3154157

[41] Scott JD, Anderson JF, Durden LA, Smith ML, Manord JM, Clark KL. 2016. Prevalence of the Lyme disease spirochete*, Borrelia burgdorferi*, in Blacklegged Ticks, *Ixodes scapularis* at Hamilton-Wentworth, Ontario. International Journal of Medical Science, 13(5), 316–324. 10.7150/ijms.14552 PMC4879763 27226771

[42] Scott JD, Foley JE, Clark KL, Anderson JF, Durden LA, Manord JM, Smith ML. 2016. Established Population of Blacklegged Ticks with high infection prevalence for the Lyme disease bacterium, *Borrelia burgdorferi* sensu lato, on Corkscrew Island, Kenora District, Ontario. International Journal of Medical Science, 13(11), 881–891. 10.7150/ijms.16922 PMC5118759 27877080

[43] Skotarczak B. 2018. The role of companion animals in the environmental circulation of tick-borne bacterial pathogens. Annals of Agricultural and Environmental Medicine, 25(3), 473–480. 30260187 10.26444/aaem/93381

[44] Tälleklint L, Jaenson TGT. 1998. Increasing geographical distribution and density of *Ixodes ricinus* (Acari: Ixodidae) in central and northern Sweden. Journal of Medical Entomology, 34(4), 521–526. 10.1093/jmedent/35.4.521 9701939

[45] Thomas JE, Staubus L, Goolsby JL, Reichard MV. 2016. Ectoparasites of free-roaming domestic cats in the central United States. Veterinary Parasitology, 15(228), 17–22. 10.1016/j.vetpar.2016.07.034 27692321

[46] Traversa D. 2012. Pet roundworms and hookworms: a continuing need for global worming. Parasites & Vectors, 5, 91–110. 22574783 10.1186/1756-3305-5-91 PMC3418564

